# Vaccines against Major Poultry Viral Diseases: Strategies to Improve the Breadth and Protective Efficacy

**DOI:** 10.3390/v14061195

**Published:** 2022-05-31

**Authors:** Rajamanonmani Ravikumar, Janlin Chan, Mookkan Prabakaran

**Affiliations:** Temasek Life Sciences Laboratory, 1 Research Link, National University of Singapore, Singapore 117604, Singapore; rajamanonmani@tll.org.sg (R.R.); janlin@tll.org.sg (J.C.)

**Keywords:** poultry viral infections, broadly protective viral vaccines, engineered viral vaccines, viral vaccine vectors, cross protection, vaccine efficacy

## Abstract

The poultry industry is the largest source of meat and eggs for human consumption worldwide. However, viral outbreaks in farmed stock are a common occurrence and a major source of concern for the industry. Mortality and morbidity resulting from an outbreak can cause significant economic losses with subsequent detrimental impacts on the global food supply chain. Mass vaccination is one of the main strategies for controlling and preventing viral infection in poultry. The development of broadly protective vaccines against avian viral diseases will alleviate selection pressure on field virus strains and simplify vaccination regimens for commercial farms with overall savings in husbandry costs. With the increasing number of emerging and re-emerging viral infectious diseases in the poultry industry, there is an urgent need to understand the strategies for broadening the protective efficacy of the vaccines against distinct viral strains. The current review provides an overview of viral vaccines and vaccination regimens available for common avian viral infections, and strategies for developing safer and more efficacious viral vaccines for poultry.

## 1. Introduction

The poultry industry is the largest and one of the most important animal protein producers for human consumption. In 2020 alone, the global consumption of poultry meat was estimated to be more than 130 million metric tons, with egg production exceeding 86.67 million metric tons. The development of high-density poultry farms due to the industry’s rapid expansion also increases the risk of disease outbreaks. Numerous etiological agents have been isolated from farmed stock, ranging from bacteria such as acute coliform bacillary and chronic tuberculosis, ecto-and endo-parasites, and fungal pathogens to at least eleven virus species that can be transmitted either horizontally, vertically, or both. Coinfection of farmed birds with multiple pathogens is common in poultry husbandry. Prior infections with avian respiratory viruses have been documented to predispose birds to secondary bacterial infection. Indeed, a study by Sid et al. [1] found increased mortality in birds co-infected with multiple viruses and *Mycoplasma gallisepticum.* Control of virus outbreaks through vaccination on poultry farms will curb the risk of zoonotic infection, limit the exchange and introduction of novel pathogens into wild stock and the establishment of a wildlife reservoir.

Viral outbreaks are one of the leading causes of economic losses for poultry industries worldwide [2]. Viral epidemics in farmed poultry negatively impacts zootechnical performances, such as feed intake, feed conversion ratio, body weight gain and egg and meat production quality. Preventive measures for disease spread include mass vaccination, surveillance and physical separation or pre-emptive culling of infected birds. In addition to circumventing economic losses, mass vaccination seeks to restrict inter-species transmission and remains one of the primary measures in disease prevention recommended by authorities worldwide [3]. Given the vast diversity of virus strains that affect poultry, current vaccination measures for early intervention and the induction of protective immunity remains inadequate. For a vaccination regimen and vaccine to be successful, numerous factors, such as farmed livestock species, vaccine types, immune status and the age of animals, have to be considered (Table 1). Despite the success of vaccines in reducing diseases, current vaccines are limited in their protection across genetically distinct strains, and the development of a broadly effective vaccine is greatly needed to combat the constant emergence of novel virus variants.

The rapid emergence of new virus variants highlights the urgent need for improved disease control and management and, in turn, drives the search for new or improved vaccine strategies. The turn of the millennium has seen the advent of a few innovative approaches to developing universal viral vaccines for humans against influenza A subtypes [4,5]. A universal or broadly protective vaccine for poultry should provide immunity against multiple circulating field strains that is also long-lasting to break the chain of horizontal transmission, a holy grail for poultry vaccinology. The present review details the current vaccines against poultry viruses of global and commercial importance, and the strategies for the development of broadly protective vaccines against the following six major poultry viral pathogens: avian influenza virus (AIV), Newcastle disease virus (NDV), Marek’s disease virus (MDV), infectious laryngotracheitis virus (ILTV), infectious bursal disease virus (IBDV) and infectious bronchitis virus (IBV).

## 2. Types of Virus Vaccine Platforms for Poultry

Among the different vaccine platforms detailed in this section, experimentally proven vaccine technologies such as live and inactivated vaccines have a long history of use on commercial farms as a prophylactic against economically important poultry viral diseases with global significance.

### 2.1. Inactivated Virus Vaccines

The culture of native seed virus in embryonated chicken eggs (ECE) or in cell cultures is one of the oldest methods of vaccine preparation. Cultured viral particles are typically inactivated via physical (e.g., ultraviolet or gamma radiation and heat) or chemical (e.g., formalin, β-propiolactone, or binary ethyleneimine) means to destroy infectivity while preserving immunogenicity. Inactivated vaccines are considered a safer alternative to live vaccines due to their inactive status. However, inactivated viral vaccines are low in immunogenicity and require booster doses and formulation with adjuvants such as oil, saponins and aluminum hydroxide for longer lasting immunity. Immune responses triggered by inactivated vaccines typically consist of humoral immunity with a slow onset period and are unsuitable for a DIVA strategy [6]. In addition, protection is prevented by pre-existing MDA in young animals. Above all, administering inactivated vaccine through intramuscular injections is a laborious process and ill-suited for high-density farming [7].

### 2.2. Live Attenuated Vaccines

Live attenuated (LA) vaccines have a long history of use against several poultry diseases. Conventional attenuation is typically achieved through serial passage of the wildtype (WT) virus in either irrelevant cell culture or host. However, the attenuation process requires a long turnaround time. Production of LA vaccine is cost-effective owing to minimal scale-up and downstream processing costs. Generation of LA vaccines by reverse genetics (RG) can bypass laborious conventional methods for developing attenuated strains. Attenuated viruses are generated from an infectious clone and engineered in vitro through RG, which is a tractable and rapid approach [8,9,10]. Other advantages of live virus vaccines lie with their ability to induce both humoral and cellular immunity and their ease of administration via drinking water, spray or in ovo. On the other hand, the risk of reversion to ‘WT’ or recombination with circulating pathogenic strains still exists.

### 2.3. Subunit Vaccines

The whole pathogen is not essential to confer complete protection against the disease, and fractionating virus proteins in chemically defined formulations has been one of the regulatory norms for minimizing side effects from vaccine components other than the immunogen. Recombinant subunit protein vaccines are among the most well-established, stable and DIVA compatible [6]. Regardless, the major disadvantages of subunit vaccine are the relatively low yield and complex purification process that can result in high manufacturing costs. As a recombinant protein, a subunit vaccine possesses low immunogenicity and requires high dosage, frequent boosters and adjuvants to enhance the protective response [11].

### 2.4. Virus-Like Particles Vaccines

VLPs are structural proteins with morphological features that resemble virus structures. Due to the similarity in structure, VLPs have successfully been utilized as novel vaccines against several viral pathogens. VLPs can be assembled using either prokaryotic or eukaryotic expression systems. Experimental studies have shown VLPs to confer high levels of protection against viral infections such as infectious bronchitis virus and avian influenza in chickens. The utilization of VLPs as a vaccine is considered a safer alternative to inactivated and attenuated vaccines due to the lack of genetic material. The safety profile, immunogenicity, protective efficacy and host immune response have also been comprehensively described [12]. In addition, VLPs can activate dendritic cells, which are crucial for stimulating innate and adaptive immune responses. Nonetheless, the high cost of expression and purification, cold chain requirement and stability in field conditions limit their use for commercial application. Production of VLPs in plant-based expression systems offers potential advantages in increased safety and scalability at a low cost [13].

### 2.5. Nucleic Acid Based Vaccine

Another approach to vaccination is through the use of DNA vaccines. DNA vaccines can be engineered to encode genes for the expression of a specific or multivalent antigen in the transfected host. DNA vaccines elicit both cellular and humoral immunity and can be designed to trigger specific cell mediated immune (CMI) responses to facilitate lymphoproliferation and cytokine secretion. Nevertheless, DNA vaccines come with their own set of challenges. The first is the difficulty in integration of the DNA vaccine [14] into the host genome, and the second is the selection of suitable adjuvants for the formulation. The presence of antibiotic resistance genes in the plasmids also carries the risk of transferring drug resistance to bacteria present in farmed birds. A variation of the DNA vaccine is the mRNA-based vaccine. Experimental vaccination studies with the HA2 and M2e antigens from H9N2 was shown to elicit a broad spectrum of protection against AIVs and could potentially be used as a novel and broadly protective vaccine against influenza viruses [15]. However, the disadvantages of mRNA lie with its requirement for low-temperature storage and formulation with expensive adjuvants such as chitosan.

### 2.6. Recombinant Virus Vector Vaccines

Viruses such as fowl pox virus (FPV), turkey herpesvirus (HVT), adenovirus, ILT and MDV are established recombinant viral vectors used in the production of poultry viral vaccines [16,17]. Among the commercially approved recombinant virus vector poultry vaccines, two of the most commonly used viral vectors are an attenuated fowl pox virus (FPV) [18] and turkey herpesvirus (HVT) [19]. Recombinant HVT (rHVT) and FPV (rFPV) vaccines are phenotypically stable, do not revert to virulence and are rarely transmitted horizontally [20]. Furthermore, these recombinant vaccines can be conveniently administered in ovo or by subcutaneous injection at one day of age [21,22]. Current third generation vaccines also offer the advantages of well-defined mutations for virulence attenuation and regulated expression to identify the location of expressed antigen. Past vaccines have primarily utilized adenovirus [23], HVT and pox viral vectors, but recent focus has shifted towards HVT-based vectors as a result of their higher efficacy. Disadvantages with the use of virus vector vaccines are the need for individual vaccination and interference of vaccine efficacy by MDA. Lastly, recombinant viral vector can be modified to accommodate immunogens from two different pathogens for broadening the scope of protection. An example is the successful incorporation of IBD and ND (Vaxxitek^®^ and Innovax^®^) and IBDV and ILTV (Farmune^®^-HVT-IBD-LT) into rHVT for the production of trivalent vaccines [16,24].

Recent advances in genome editing using CRISPR-Cas9 (clustered regularly interspaced short palindromic repeats associated RNA-guided endonuclease) have paved the way for developing multiplex rHVT vector poultry vaccines. CRISPR-Cas9 can be employed for targeted mutagenesis of viral vectors such as HVT [25,26,27], ILTV [28] and duck enteritis virus (DEV) [29] to express multiple antigens against viral diseases such as NDV [28], AIV [27,29], MDV [25,26], and ILTV [26] concurrently. Additionally, CRISPR can be complemented with other strategies, for example, the error-prone non-homologous end joining (NHEJ)-CRISPR/Cas9 system and cyclization recombinase (Cre)/locus of crossover (Lox) system [30], to create a novel NDV-ILTV recombinant vaccine vector with better safety features and higher efficacy [28]. Moreover, CRISPR/Cas9 is a relatively quick, low-cost and very precise way to target and edit specific genetic sequences to generate novel multivalent viral vector vaccines. The few concerns over the technology are possible off-target mutations in host cells and the consequences of such mutation.

Several viral vectors have been developed to express heterologous antigens in vitro as well as displaying them on the viral surface. Among these, baculovirus (BV), an insect pathogen, has been widely used as a tool for protein production in insect cells and is an excellent tool for displaying foreign proteins on the viral envelope [31]. Advantages of the BV system include higher vector construct stability, high production yield and eukaryotic-like post-translational modifications. The vector can also be modified to accommodate multiple promoters and a large genome to produce complex proteins such as VLPs. Detailed reviews of the methods, applications and current modification strategies have been discussed previously [32,33,34,35]. However, antigens produced by the BV-insect cell expression system are expensive to purify and require adjuvants for improved immunogenicity. Alternatively, the targeted protein can be expressed on the baculovirus surface (BVS) and used as a vaccine. BV was found to have strong adjuvant properties, and can induce both humoral and CMI responses against the vaccine immunogen [36]. BVS antigens can make baculovirus an efficient vaccine vehicle with enhanced protective immunity against the displayed antigens in animal models [31].

## 3. Major Poultry Viruses: Past and Current Vaccine Strategies

### 3.1. Avian Influenza Virus (AIV)

Avian influenza virus is one of the most contagious viruses in farmed poultry and a significant concern due to its zoonotic potential. The poultry pathogen influenza A (Type A) is a segmented, negative-sense RNA virus. Influenza A viruses are categorized into different subtypes depending on their surface antigens known as hemagglutinin (HA) and neuraminidase (NA). Eighteen different subtypes of HA have been documented along with a total of eleven subtypes recorded for neuraminidase (NA) antigens. Combining the two antigens could theoretically produce 198 subtypes, most of which have already been identified in nature [37]. Phylogenetically, all mammalian influenza viruses are derived from the avian influenza viruses [38]. AIV is classified into two pathotypes known as highly pathogenic avian influenza (HPAI) and low pathogenic avian influenza (LPAI) based on the severity of disease produced in gallinaceous poultry. HPAI occurs in avian hosts with high morbidity and mortality whereas infection with LPAI results in variable morbidity and low mortality. According to the World Organisation for Animal Health (OIE, Office International Des Epizooties), HPAI viruses and LPAI H5 and H7 infections are notifiable diseases due to their highly transmissible nature. LPAI has a global distribution with regional variations in type and the targets of infection. The H9N2 subtype is an example of a LPAI pathotype affecting poultry worldwide, and is endemic to Asia, the Middle East and Africa [39,40,41]. Another LPAI, H7N9 was identified as the cause of several outbreaks in China but has not been isolated in other parts of the world. H7N9 started off as an LPAI in the first four waves of the outbreaks but has since acquired characteristics of an HPAI in the fifth wave, including a polybasic cleavage site, conferring upon it a high potential for zoonosis [42]. Developed countries, with stringent measures of biocontainment, had eradicated HPAI from all commercial poultries. The Asian HPAI H5N1 strain is prevalent in the wild birds of Eurasia and poultry of Asia and the Middle East, but only been reported in the US and Australasia from 2016. Most recently, H5N1 has been reported in both wild birds and poultry in the East coast of Canada in 2021 and the US in 2022 [43]. The lineage of the current circulating H5 HPAI can be traced to Asia and more specifically the goose/Guangdong lineage (Gs/GD) in 1996. Since then, several outbreaks of a new lineage of the same virus have swept through Asia, the Middle East, Africa and Europe from 2003, resulting in a massive culling of birds. The wave resulted in the virus is now considered to be endemic in the poultry of Asia and Africa, with the Gs/GD lineage diversified into several genetic clades and multiple subclades.

Protection against avian influenza AIV is primarily mediated by the humoral immune response, which produces neutralizing antibodies against the HA and NA antigens. However, immune responses triggered by both antigens are not considered to be broadly protective. Additionally, the cell mediated immune response involving cytotoxic T-lymphocytes is known to play a vital role in clearing infection and providing protection between antigenic distinct AIV [44,45].

#### 3.1.1. Conventional AIV Vaccines of Poultry

Modern poultry AIV vaccines are primarily developed with platforms such as inactivated WT or RG; subunit or virus-like particles; recombinant virus vectors; DNA vaccines; and defective replicating alphaviruses, i.e., defective Venezuelan equine encephalitis (VEE) expressing HA protein or a DNA vaccine with a H5 AI virus gene insert. Currently, there is no universal vaccine against AIVs for poultry [46]. A comprehensive compilation of seed strains or subunit sources has previously been evaluated [47,48]. AIV is a non-eradicable zoonosis due to rapid antigenic drift and multiple virus reservoirs in wildlife [49]. To date, 95% of the AIV vaccines in use are made up of inactivated whole AIV vaccines grown in ECE with the LPAI virus as seed strains [50].

The LPAI was later replaced by HPAI virus seeds (H7N3 or H5N1 Gs/GD lineage), but the highly demanding biocontainment and stringent quality control that comes with the use of an HPAI are major hurdles for their use [46]. Nonetheless, introduction of RG using eight bi-directional (‘ambisense’) vector plasmids to generate influenza subtypes, especially HPAIV H5 and H7 vaccine strains [8] with a HA cleavage site matching that of LPAI, has negated the need for extensive biocontainment. In addition, the RG AIV seeds come with the added benefits of possessing six gene segments in six plasmids from the rapid-growing low-pathogenic strain of influenza A/PR8 (H1N1) and two plasmids containing the HA and NA genes from the target HPAI to produce a highly controlled 6:2 reassortant that grew in high titers in ECE [51]. More than 20 approved inactivated AI vaccines are currently in use for field application, including ‘WT’ and ‘RG’ strains of H5 and H7 subtypes [52].

Live attenuated AI vaccines (LAIV) developed from ‘WT’ strains are not recommended for poultry by the OIE/FAO due to the potential risk of reversal of the attenuated strain into an HPAI by reassortment or mutation. A stable LAIV is an attractive target since it can be formulated for needle-free mass vaccination through nasal spray. Efforts along this line resulted in the development of cold-adapted/temperature-sensitive (ca/ts) variants to serve as LAIV vaccines [53]. Interestingly, truncation of the non-structural protein 1 (NS1), a potential interferon agonist of AIV, left the latter permanently attenuated, thus ensuring its safety as a live protective vaccine [54]. One of the most promising candidates for poultry LAIV is the pc4-LAIV, an influenza virus mutant that expresses a C-terminally truncated NS1 protein due to a large internal deletion of ~190 nt in the NS gene segment. A shift in the NS1 open reading frame and a premature stop codon [55] ensures reversion to a ‘WT’ phenotype. Intranasal immunization of birds with pc-4 LAIV followed by a subcutaneous boost with an inactivated influenza vaccine, both belonging to the H7N3 subtype, showed robust mucosal antibody responses and an acceleration of seroconversion. The vaccines also exhibited a synergistic increase in serum cross-reactivity antibody titers and full protection from H7N2 heterologous viral challenge [56]. Moreover, the NS1 antibody response in a vaccinated flock conforms to a DIVA strategy for differentiation between unvaccinated and vaccinated stock. NS1-based experimental vaccine approaches are also well established and readily available [57,58]. A similar approach for creating NS1-deletion mutants has been trialed for human AIV vaccines and shown to confer protection in a mouse model [54].

#### 3.1.2. Recombinant Viral Vector AIV Vaccines

Viral vectors such as FPV, HVT, adenovirus, ILT and MDV have been evaluated as recombinant vectors for AIV vaccines [16]. Currently, five viral vector vaccines are commercially available against AIV. Viral vector vaccines for AIV include recombinant HVT-AIV, recombinant NDV-AIV and recombinant FPV-AIV. There are currently two FPV (Trovac) vector vaccines for A/turkey/Ireland/1378/83 (H5N8) and A/chicken/Guanajuato/15/(H7N3), and two HVT (Vectormune) vector vaccines for A/Swan/Hungary/06/(H5N1 clade 2.2) and A/chicken/Guanajuato/15/(H7N3) available for subcutaneous injection that induce hemagglutination inhibition (HAI), neutralizing antibodies and protect against clinical signs and mortality in vivo [16]. A heterologous booster with an inactivated H5 AIV dose is recommended with AIV and FPV vectors after 2–3 weeks of age to reduce the effects of MDA [17,59]. The other common recombinant virus vector is NDV, an avian virus with a single-stranded negative-sense RNA belonging to orthoavulavirus type 1 (AOAV-1) serotype 3. The first study using NDV as a vector to carry AIV A/WSN/1933 was published in 2001 [60], and since then, multiple AIV vaccines have been formulated using the rNDV vector, mostly as bivalent vaccines to induce immunity against NDV and AIV in poultry. A lentogenic rNDV vector was similarly developed to target the H5 and H7 antigens [61,62]. The major disadvantage of rNDV-based vaccines is the interference of anti-NDV MDA, which reduces the efficacy and longevity of protection [63]. CRISPR/Cas9 technology has been utilized to create vaccines against specific AIV subtypes. One study employed CRISPR/Cas9 for targeted genome engineering of a trivalent vaccine using the DEV vector to include the H5 from H5N1, pre-membrane protein (PrM) and envelope glycoprotein (E) of the duck tembusu virus (DTMUV) to protect against H5N1, DEV, and DTMUV in ducks [29]. In other work, genes encoding structural proteins of the alphavirus VEE vector were replaced with the transgene of interest. RNA replication of the gene of interest was then observed to stimulate antigen-presenting cells in the lymphoid tissue to generate a robust immune response [64]. Among the two VEE replicon-based AIV vaccines, a vaccine expressing HA from H5N8 (RP-H5) has received a conditional USDA license [65].

The BV system is another alternative for the development of avian-based influenza vaccines, adapted from the established model for human influenza vaccine production [66,67,68]. This versatile platform serves to produce recombinant protein antigens from AIV [69], first as subunit vaccines, and later as VLPs [68]. Following the large-scale implementation of the recombinant vector-based vaccine strategy in the early 2000s, a recombinant BV encoding HPAI and NDV antigens was expressed in insect cells and used as an inactivated, oil emulsion-based vaccine prototype for commercially approved poultry vaccine in Egypt and Mexico. The results were replicated in another two studies with rBV vaccines promising cross clade protection [70] and protection against lethal heterologous challenge [66]. Since BV expression protects the native conformation and biological activity of hemagglutination, multiple HAs have been co-expressed in BV-secreted VLPs to elicit a protective immune response in ferrets against H5, H7 and H9 [71]. Expression of HA trimers, co-expression of multiple homologous proteins [72] and heterologous AIV subtypes in a VLP subunit vaccine are possible with the BV system for protection against LPAI [68]. In addition, there are increasing reports on the application of the BV system for surface expression of HA along with other heterologous protein antigens [72,73]. The efficacy of surface-displayed HA-based BV vaccines for AIV H5 [74], H6 [75], H7 [76] and H9 [77] subtypes has been evaluated for intranasal [76] and oral routes of administration [78]. Further advancements will be made by using hybrid promoters for the enhanced expression of proteins on theBVS and including a molecular adjuvant in the vector to boost immunogenicity of the antigens displayed on the BV. If adapted for an oculo-nasal or oral formulation, multiple heterologous immunogens displayed on the BV will be one of the most promising strategies for the development of a universal or broadly protecting poultry vaccine.

#### 3.1.3. Nucleic Acid Based AIV Vaccines for Poultry

The US Department of Agriculture (USDA) conditionally approved the first DNA avian flu vaccine against H5N1 for chickens in 2017 [79,80]. Plasmid DNA containing the H5 gene with either NA, M or NP genes was formulated with Lipofectin^®^ or cationic derivatives trimethylpolyprenylammonium iodides (PTAI) and the Esat-6 gene from *Mycobacterium tuberculosis* as an adjuvant and utilized for immunization against H5 influenza virus in chickens [81,82]. Protection against H5 and H7 HPAI was observed after vaccination with a mixture of plasmids encoding the HA from both subtypes. Intriguingly, the NA or the more conserved NP of the matching subtype rendered only partial protection. Since the immunogenic NP is not required for protection, and hence, not necessarily part of the vaccine, antibodies against NP can be used as a marker for DIVA.

### 3.2. Newcastle Disease Virus (NDV) and Vaccination Strategy

NDV is one of the most important contagious diseases affecting the respiratory, nervous, and digestive systems of poultry worldwide, resulting in severe losses to the poultry industry. The virulence, pathotypes, disease manifestations and its panzootic potential in poultry are well described in the literature [83]. The major challenge for NDV vaccine development is the constant evolution of genetically diverse genotypes with wide-ranging geographical distribution. NDV has been documented to produce a total of 18 genotypes with more than 236 avian species as susceptible hosts [83,84,85,86]. The major viral surface glycoprotein, hemagglutinin-neuraminidase (HN), is responsible for host cell receptor binding and possesses neuraminidase activity to prevent virus self-aggregation to promote cell-virus or cell-to-cell fusion. HN is also immunogenic and elicits neutralizing antibodies in the host during infection. NDV was first discovered in an outbreak in Newcastle upon Tyne in the UK. Subsequent outbreaks were traced to Java, Indonesia and Korea in 1926. Between 1926 and 1981, four ND panzootic strains were recorded worldwide and currently, in all African countries. Apart from being panzootic, ND is native to Asia, Africa, the Middle East, Central and South America, and sporadically found in Europe [87,88]. Protection of poultry from NDV is provided by commercially available vaccines, mainly live vaccines (either attenuated or vectored vaccines) and inactivated vaccines.

Live attenuated vaccines developed from lentogenic NDV strains, especially LaSota or inactivated oil emulsion vaccines [89], are commonly employed in NDV vaccine formulations. However, most live attenuated NDV LaSota vaccines (or other genotype II derivatives) can cause respiratory symptoms. Thus, mass vaccination by spray or drinking water with live attenuated ND vaccines is used to minimize vaccination reactions. Live NDV vaccines provide a short duration of protection, and frequent vaccine failures are reported due to pre-existing conditions, such as immunosuppression due to co-infection or interference of anti-NDV MDAs in young birds [90]. Live vaccines can be complemented with an inactivated NDV booster to induce long-lasting humoral antibody responses.

There are at least 13 different recombinant viral vector vaccines licensed commercially to protect poultry against NDV. The first generation consisted of two FPV vectors, followed by six second generation and five third generation vaccines incorporating an HVT vector with the Fusion (F) protein or the hemagglutinin-neuraminidase (HN) protein of NDV. Most of these vaccines are administered via subcutaneous routes and in ovo [16,24]. However, a downside to the use of HVT vectors is interference and neutralization between HVT vaccines expressing different immunogens. Instances of immune interference have been documented after avian vaccination with an HVT in ovo when existing antibody neutralized a second HVT vector vaccine post-hatch [91]. NDV is commonly used as a viral vector to express two heterologous proteins simultaneously for the creation of bivalent vaccines [92,93] such as HPAI H5 [61,94] or H7HA [95]. NDV is a natural pathogen of birds that possesses a small modular genome that ensures delivery of the immunogen to targeted tissues to elicit mucosal and systemic immunity [96]. Bivalent vaccines have been generated for IBDV, IBV, ILTV and avian metapneumovirus [97]. Apart from being cost-effective, these bivalent vaccines are highly stable and replicate well in vitro [97] and in vivo [95]. An NDV-AIV vaccine was commercialized for field application in China and Mexico with more than 11.7 billion doses administered during 2006–2012 [98]. A thermostable rNDV bivalent vaccine against HA and NDV Fusion protein has been demonstrated to induce a higher HI antibody titer that significantly reduced virus shedding in immunized animals challenged with H9N2 AIV and NDV [99]. There are currently over 11 different experimental bivalent vaccines utilizing rNDV as the vector backbone for IBDV, ILTV, IBV and AIV. Most recently, a 15 residue (242–256aa) linear immunodominant epitope (IDE)-4 was identified in the hemagglutinin-neuraminidase (HN) protein of Newcastle disease virus with potential to be adapted as an epitope-based vaccine [100]. The different properties of live, inactivated and vectored ND vaccines are compared in detail by [85].

### 3.3. Marek’s Disease Virus (MDV) and Vaccination Strategy

The etiological agent of Marek’s disease (MD) is an alphaherpesvirus belonging to the genus Mardivirus, in the family Herpesviridae. MDVs are classified into three different species based on their serotypes: Gallid herpesvirus 2 (GaHV-2, MDV serotype 1 or MDV-1), Gallid herpesvirus 3 (GaHV-3, MDV serotype 2 or MDV-2) and Meleagrid herpesvirus 1 (MeHV-1, MDV serotype 3, MDV-3 or HVT) [101]. MDV-1 comprises of oncogenic viruses of variable virulence, whereas MDV-2 consists of non-oncogenic viruses from chickens. Lastly, MDV-3 is made up of non-oncogenic viruses from turkeys [102]. Recently, MDV has been demonstrated in lymphomatous tumors in commercial turkeys in several countries [103]. At present, three different serotypes of MDV vaccines are licensed for use in poultry. The MDV-1 serotype vaccine includes MDV-1 strain CVI 988, a mildly pathogenic and attenuated strain [104]. The first case of MD was documented in the US in 1914 soon to be followed by various countries in Europe. Subsequent outbreaks of MD in 1922 resulted in increased morbidity and mortality of 20% in farmed birds. A dedicated laboratory was eventually established in Michigan during the late 1930s to study MD [105]. Attenuated MDV-1 vaccines were traditionally developed through passage in cell culture with the introduction of random mutations in the viral genome [106]. The first MD vaccine, HPRS-16/att, was generated by serial passage of a virulent MDV in chicken kidney cells and provided protective immunity against a challenge with virulent MDV [107]. HPRS-16/att was soon replaced by CVI 988, the current global gold standard [108].

The MDV-2 serotype vaccine includes the SB-1 strain [109] or 301B/1, both of which are naturally apathogenic and have been utilized as templates for creating a bivalent HVT recombinant vaccine [110,111]. However, due to a lower protection against very virulent (vv) MDV and interference by MDA, MDV-2 is generally used in combination with other serotypes and are components of bivalent or trivalent vaccines [112].

The MDV-3 serotype vaccine consists of another apathogenic strain, MDV-3 (HVT) FC126 cloned into an HVT vector [113]. The HVTs are non-oncogenic viruses from turkeys and are antigenically related to oncogenic MDV-1. HVT has been shown to protect birds against MDV challenge and was officially licensed for use in the US in 1971 [114]. Since then, HVT is widely used as a monovalent vaccine against MD for broilers or as part of a polyvalent vaccine for breeders and layers [115]. The most commonly used HVT strain is FC126. Preparation of MDV-1 and -2 vaccine strains requires special handling and storage inside infected cells. However, unlike the MDV-2 and MDV-1 serotypes, MDV-3 is available as a cell-free vaccine prepared by the sonication of HVT-infected cell cultures [116]. Nonetheless, use of MDV-3 as a vaccine suffers from low efficacy due to MDA [117]. Although MD vaccines are effective at protecting chickens against tumors and mortality, they do not provide sterilizing immunity, and vaccinated chickens are still susceptible to infection. The widespread use of MD vaccines is considered the driving cause for the evolution of MDV field viruses towards greater virulence [118] with a drastic change in MDV for central nervous system tropism, resulting in higher mortality. Classification of MDV has also been updated to reflect the pathogenicity of the strain, ranging from mild (mMDV), virulent (vMDV), very virulent (vvMDV) to very virulent plus (vv + MDV) [102]. Hence, there is a current urgent need to develop a next generation MDV vaccine that can induce sterilizing immunity to prevent infection and disease transmission.

### 3.4. Infectious Laryngotracheitis Virus (ILTV) and Vaccination Strategy

ILT is an avian respiratory disease caused by a Gallid herpesvirus-1 (GaHV-1) belonging to the genus Iltovirus, and the subfamily Alphaherpesvirinae within Herpesviridae family. Infection with ILT is associated with decreased egg production, weight loss and mortality, and the transmission of disease typically occurs through aerosols and fomites [119]. The special Committee on Poultry Diseases of the American Veterinary Medical Association coined the term infectious laryngotracheitis in 1931. ILT is prevalent in the United States, Europe, China, Southeast Asia and Australia [105]. There are two types of modified live ILT vaccines. The first is a live strain attenuated by passage in embryonated eggs [120], whereas the second was attenuated by serial passage in tissue culture [121]. Both vaccines are effective in chickens [122] and are widely used worldwide. However, ECE-based ILT vaccines are associated with adverse effects due to their reversion to virulence.

ILT recombinant vaccines currently available for commercial use utilize either FPV or HVT vectors with expression of an array of ILT genes, such as gB and UL32 (Vectormune^®^ FP LT + AE, CEVA Biomune), gI and gD (Innovax^®^ ILT, Merck Animal Health) or gB and UL-32 (Vectormune^®^ HVT LT, CEVA Biomune) [123]. In addition, an NDV-LaSota strain expressing ILTV glycoproteins [124], modified vvMDV expressing ILTV glycoproteins [125] and recombinant vaccines expressing different ILTV glycoproteins, including gB, gC, gD, gG, gI, gJ, TK, UL0, UL32 and UL47, have also been introduced and evaluated. Another NDV vector generated to express gD for protection against ILT and NDV and was also found to be genetically stable [126]. Additionally, a recombinant HVT vector (HVT-NDV-ILT) expressing the F gene from NDV and the gD and gI genes from ILTV was found to induce 97%, 94% and 97% protection against velogenic NDV (GBTexas), ILTV (LT 96-3) and MDV (GA 5) strains, respectively [127].

The advantages associated with the use of recombinant vaccines includes the inability of the live strain to transmit between chickens, recombine and revert to the wildtype virulent form [93,128]. A bacterial artificial chromosome (BAC) with genes encoding gB or gJ from ILTV was introduced into a ‘meq’ gene-deleted vvMDV vector to create two different vaccine strains known as BACDMEQ-gB and BACDMEQ-gJ. The vaccines conferred protective immunity upon GaHV-1 challenge to provide comparable protection as a commercial ILT-HVT vector vaccine [128]. Both BACDMEQ-gB and BACDMEQ-gJ were found to induce rapid immunity against an early vv + MDV challenge to surpass the protection provided by the GaHV-1 HVT vaccine and the serotype 1 attenuated CVI 988 vaccine. The BACDMEQ vector was also effective in preventing MD-induced tumors and immunosuppression [129].

Numerous immunization routes have been evaluated for ILT vaccines, with the most practical route being in ovo vaccination of broilers with HVT, MDV, or the GaHV-1 ‘meq’ gene-deleted vector. Layers can be vaccinated at day 1 of age with HVT, MDV, FPV, NDV vector vaccines or GaHV-1 ‘meq’ gene-deleted strains followed by vaccination with live attenuated ILT strains [128]. Administration of VLPs with gB or gG in ovo was found to induce a humoral response with no adverse side effects. A recombinant HVT-ILT administered in ovo was however ineffective at breaking the chain of viral transmission [130], but vaccination with eye drops was successful in stimulating the conjunctiva-associated lymphoid tissues (CALT) and the Harderian gland (HG) to provide immunity against ILT [131]. A comprehensive list of registered and experimental vaccines against ILTV has been previously reviewed [129].

### 3.5. Infectious Bursal Disease Virus (IBDV) and Vaccination Strategy

IBD, or Gumboro disease, is a highly contagious immunosuppressive viral disease of young chickens. The etiological agent of IBD is a non-enveloped, double-stranded bi-segmented, A and B, RNA-bearing avibirnavirus belonging to the family *Birnaviridae*. Infection with IBDV results in clinical symptoms of depression, diarrhea, dehydration and neoplasia of the bursa of Fabricius. IBD outbreaks have resulted in high morbidity with significant economic losses worldwide. VP2 is a structural polypeptide of IBDV and the major IBD antigen targeted for vaccine development [132]. IBDV has two recognized serotypes (serotypes I and II), but only serotype I is found to be pathogenic in chickens. Recent outbreaks with high mortality have been largely associated with novel and re-emergent IBD phenotypes known as variant IBDV (VarIBDV) and very virulent IBDV (vvIBDV). In the 1980s, vvIBDV was responsible for outbreaks in the Netherlands, Africa, Asia and South America with a mortality of 90%. Subsequently, strain DV86 was reported in the UK, Japan and Belgium. Most countries, including Central Europe, Russia and the Middle East, have reported cases of acute IBDV [132]. There are currently seven genogroups based on the VP2 classification. Among the various genogroups, only genogroup 1 is found globally [132].

Protection against IBD is achieved through a combination of passive MDA inherited from immunized parent stock and active immunization of chicks with modified live vaccines (MLVs). Most IBD MLVs originated from attenuated strains of IBDV serotype 1. In the late 1990s, immune complex vaccines containing live intermediate plus strains were mixed with hyperimmune sera against IBDV to produce virus-antibody complex vaccines that were administrated on day 1 or in ovo. The presence of high levels of anti-IBDV MDA does not interfere with protection against vIBDV and vvIBDV [133]. MLVs are classified as mild, intermediate, or intermediate plus based on their attenuation level and their ability to break through MDA. Milder MLVs are effective vaccines but remains highly sensitive to interference by MDA. Intermediate MLVs retain residual virulence, causing bursal atrophy with consequent immunosuppression. In parent stock, inactivated IBDV vaccines are administered before the onset of lay to boost the immune response elicited by MLVs, thereby increasing the level of MDA that is transferred to the progeny [134]. As the progeny originate from diverse parent stocks of different ages, it is difficult to achieve the same level of MDA in all progenies. Hence, the vaccination regimen must be optimized for time, dosage and frequency of immunization of parent stocks using tools like ‘the Deventer formula’.

Besides live vaccines, several commercial recombinant vector IBDV vaccines have also been developed. However, the first generation, employing an FPV vector, was found to provide low protection. Currently, two second generation rHVT-IBD vaccines with host protective VP2 adapted from Faragher 52/70 (Vaxxitek HVT + IBD) and variant Delaware E (Vectormune IBD) are available. Third generation rHVT vaccines, Innovax IBD-ND and Ultifend IBD ND, were similarly formulated to include the VP2 immunogen from IBDV and the F gene from NDV. Another combination is a trivalent vaccine (Ultifend ND IBD) against IBDV, NDV and MD. Two more vaccines were produced and licensed in 2020 using a rHVT vector (Vaxxitek HVT + IBD + ND and Vaxxitek HVT + IBD + ILT) containing VP2 from IBDV, F from NDV genotype VII 1.1 and gD from ILTV USDA. The vaccines have been optimized for administration in ovo or via the subcutaneous route to provide long-lasting immunity. Another experimental study successfully displayed VP2 (BV-VP2) from IBDV on the BV viral surface. Vaccination of chickens with the BV-VP2 vaccine elicited high levels of VP2-neutralizing antibodies and stimulated antigen-specific lymphocyte proliferation. A challenge experiment of BV-VP2 vaccinated chickens with vvIBDV HZ strain was found to reduce clinical signs of disease and mortality [135]. The use of CRISPR/Cas9 technology has yielded the first rHVT multivalent vaccine (rHVT/IBD/ILT/AI H9) with three different expression cassettes (IBDV VP2, ILTV gD–gI and AIV H9 HA) to create a potential vaccine for simultaneous protection against ILTV, IBDV, AIV and MDV [26].

### 3.6. Infectious Bronchitis Virus (IBV) and Vaccination Strategy

IBV is a highly infectious, positive sense, single-stranded, enveloped RNA gammacoronavirus from the *Coronaviridae* family [136]. Outbreaks of IBV can impose significant economic losses on high-density commercial poultry farms, with up to 80% mortality. IBV was first identified in the United States in 1930, and the Massachusetts (Mass) strain was the earliest strain to be identified and remains one of the most common IBV strain encountered globally. Since the 1950s, IBV strains have been isolated in Africa, Asia, India, Australia, Europe and South America [105,136]. Genomic analysis of spike protein S1 sequences in IBV has revealed 7 genotypes and 35 lineages [137]. Surveillance of farms often reveals co-circulation of multiple IBV serotypes with low cross-protection from the vaccine strain against non-related field strains. Moreover, endemic IBV strains differ across geographical regions, complicating global disease control strategies. Thus, there is a current dire need for the creation of a novel, broadly protective IBV vaccine that can provide long-lasting sterilizing immunity.

The current approach to commercial vaccination typically takes the form of immunization with two antigenically diverse IBV variants selectively complemented with an inactivated vaccine for layers [138]. Commercial IBV vaccines are generally administered early in life starting with day-old chicks [136]. Protection of farmed stock against IBV is currently undergoing a gradual shift from the use of a generic strain to the identification and production of IBV vaccines against the dominant circulating field strains, but vaccinating against a specific field strain is ineffective for cross-protection between unrelated IBV strains [139]. The design of broadly protective IBV vaccines typically falls into two categories with immunization utilizing either whole or part of a protectotype IBV strain such as Massachusetts (Mass), or whole or part of a recombinant chimeric IBV strain comprising antigenically distant strains to widen protection.

Vaccine candidates typically target the four major virion assembly structural proteins known as spike (S), membrane (M), small envelope (E) and nucleocapsid (N) [139]. Among the four proteins, the S protein is the most immunogenic and frequently exploited for the creation of IBV vaccines. The search for an efficacious and cross-protective IBV vaccine has yielded a few conventional and novel IBV vaccines, among which immunization with an ‘RG’ chimeric IBV strain and VLPs elicits the best immune responses and is the most promising technique for creating a broadly protective IBV vaccine.

Inactivated IBV vaccines used affinity-purified IBV antigens or formaldehyde or β-propiolactone-inactivated viruses, and in particular, the Mass-type (GI-1) genotype for immunization. The use of multivalent inactivated vaccines containing Mass antigens in association with live vaccines was shown to boost antibody titers and enhance protection against two homologous and three antigenically distinct heterologous IBV strains [140]. A study comparing an inactivated IBV BR-1 vaccine with two different adjuvants—chitosan nanoparticles (CS) and Montanide ISA 71 oily adjuvant (OA)—revealed that a single dose of the vaccine with CS was sufficient to achieve complete protection when compared to OA [141]. Similarly, resiquimod, a TLR7-agonist, stimulates secretory IgA production and increases antigen-specific humoral responses and CMI when formulated with an IBV vaccine [142].

Live IBV vaccines are notably superior to inactivated vaccines [143]. Cross-protection studies [144] have found that animals vaccinated with two doses of an attenuated Mass vaccine are cross-protected against heterologous virus challenge [144,145,146].

IBV recombinant vaccines generally adopt immunogens such as the S1 and S2 subunit of the spike protein to create subunit vaccines. Immunogens are commonly purified from cell lines or expressed by baculovirus vectors [147]. Several studies have found S1 to be insufficient for cross-protection against a heterologous IBV challenge [148,149] but its protective efficacy was greatly enhanced when paired with other IBV surface proteins, such as the N (nucleocapsid) or M (membrane) proteins. Vaccination of pathogen-free birds with a recombinant baculovirus expressing both S1 and N induced higher levels of antibody and CD4+ and CD8+ T-cells when compared to birds vaccinated with mono-antigen vectors [150]. A study by Eldemery et al. [151] similarly found a significant reduction in viral load in challenged animals following immunization with recombinant IBV S1 and the ectodomain of S2.

Another potential path of control for IBV is with VLPs. An advantage of using VLPs over live IBV vaccines lies with its inactive status. VLPs consisting of S and M protein were found to produce functional antibody levels comparable with animals vaccinated with inactivated H120 [152]. Most importantly, the S and M VLPs overcame the low cellular signature response of a recombinant vaccine to stimulate cellular immunity [152]. A VLP constructed with S, M and E protein of IBV has likewise been demonstrated to elicit high neutralizing antibodies, and higher IL-4, IFN-γ and secretory IgA (sIgA) in comparison with groups vaccinated with dual antigen-VLPs. Protective coverage from IBV VLPs can also be enhanced with foreign viral antigens such as neuraminidase from avian influenza H5N1 virus [153].

Chimeric recombinant and novel bivalent viral vector vaccines created through ‘RG’ are ideal for creating vaccines with dual protection against IBV and an alternative viral pathogen. RG vaccines require a shorter production process and have improved safety profiles to circumvent recombination events when used in the field. Ellis et al. [154] found that a full spike protein provides better protection than the use of heterologous S1 and S2 when encoded into a chimeric avirulent BeauR IBV strain. Investigation into the expression of a S1 protein from a virulent isolate in a lentogenic IBV strain has also been successfully used as a chimeric vaccine to provide protection against infection by the wildtype virus [145]. Investigation into the expression of an S1 protein from a virulent isolate in a lentogenic IBV strain has also been successfully used as a chimeric vaccine to protect against infection by the wildtype strain [155]. Various bivalent recombinant vectors have been created by splicing the IBV S protein gene or its subunits into a variety of viral backbones ranging from NDV [156], duck enteritis virus [157], MDV and avian metapneumovirus [158]. Among them, the NDV vector harbors the most potential as a widely protective IBV vaccine. NDV is a well-established expression cassette for the development of human and avian recombinant viral vaccines [159,160]. Monovalent [161] and multivalent DNA vaccines combining S1, M and N plasmids were effective at inducing cellular immunity against IBV. A DNA vaccine could also be enhanced by formulation with novel delivery vehicles, such as chitosan and saponin formulated nanoparticles [162], co-expression of an IL-2 gene [163] or granulocyte-macrophage colony-stimulating factor (GM-CSF) gene [163].

The viruses discussed above are the primary pathogens of poultry; however, other viruses, such as adenovirus and reovirus, are generally considered to be secondary invaders of the upper respiratory tract of chickens [164]. Avian reoviruses (aRV) belong to the genus *Orthoreovirus*, under the family *Reoviridae*. Chickens infected with aRV usually display signs of viral arthritis (tenosynovitis), malabsorption and enteric symptoms with up to 10% mortality [165,166]. Control of aRV is through live or inactivated vaccines for breeding stock to provide passive immunity to the progeny. The emergence of novel aRV variants in America and Canada has prompted a move from inactivated and attenuated vaccines to autogenous vaccines against local circulating strains on commercial farms. Alternative vaccines against aRV have also been developed using recombinant platforms to create subunit vaccines commonly used for the creation of vaccines against other avian viruses [167].

## 4. Strategies for Broadening Vaccine Immunity

### 4.1. Neutralizing Epitopes-Based Vaccine Strategy

**Figure 1:**
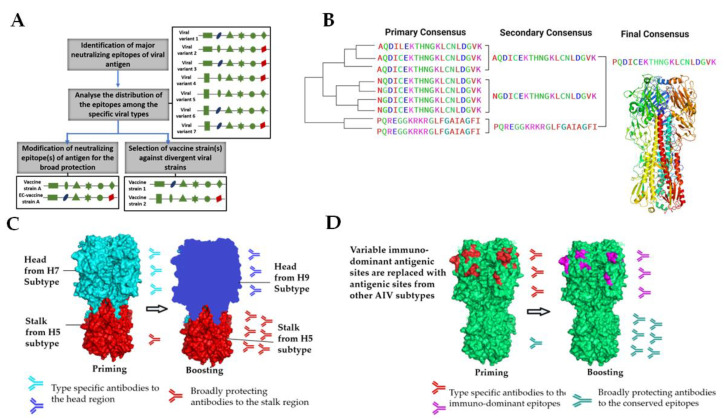
Schematic diagram of broadly protective vaccine designs. (**A**) The three major steps to the creation of neutralizing epitopes-based vaccines. Major neutralizing epitopes on the protective antigen are identified with neutralizing antibodies. Next, the distribution of the identified epitopes is analyzed across multiple lineages for the selection of optimal vaccine strains or enable the modification of a vaccine antigen to best represent the neutralizing epitopes of antigenic subtypes. (**B**) Computationally optimized broadly reactive antigen (COBRA). The consensus sequences for each genotypic group are realigned to generate a secondary consensus, which is then aligned to obtain a single final consensus sequence based on conserved regions, designated as COBRA. (**C**) The ‘chimeric’ approach involves sequential vaccination with vaccines containing HA heads of distinct influenza subtypes grafted onto a conserved HA stalk for universal or broad protection against AIV subtypes. (**D**) The ‘mosaic’ approach replaces variable immunodominant antigenic sites with equivalents from other influenza HA subtypes to produce an immunogen with conserved epitopes in both the stalk and head domains.

The protective coverage and efficacy of a vaccine can be expanded through three major steps, as specified by Prabakaran et al. [168] and shown in Figure 1A. In brief, the first step to the expansion of vaccine coverage is the identification of major neutralizing epitopes on the vaccine antigen through the use of neutralizing monoclonal antibodies (mAbs). Next, an analysis is performed to identify the variations in neutralizing epitopes among the different lineages of viral strains to enable appropriate selection of subtypes that best represent the antigenic diversity found in the viral lineages for inclusion in the vaccine formulation. This strategy was well exemplified by the same study [168], whereby three H5N1 vaccine strains (A/Vietnam/1203/04 from clade 1, A/Indonesia/CDC669/06 from clade 2.1.3.2 and A/Anhui/01/05 from clade 2.3.4) were selected based on the major neutralizing epitopes H5HA. The HA genes from these three strains were displayed individually in a baculovirus for universal protection against distinct clades of H5N1 subtype. Modified vaccinia virus Ankara was used as a viral vector to express the three selected HAs in a single recombinant construct. The constructed vaccine showed cross-protective efficacy in a mouse model and neutralization efficacy was confirmed by sero-surveillance studies of post-vaccinated guinea pig sera against multiple clades of H5N1 circulating strains worldwide between 1997 and 2012 [169].

The strategy of modifying existing neutralizing epitopes to expand protection is illustrated with an example of H5N3 (A/duck/Singapore/3/1997) modification to confer cross-protection to an antigenically diverse range of human H5N1 viruses circulating from 1997 to 2004. The original H5N3 HA epitopes located at the 140th loop and 190th α-helix were modified to encompass the H5N1 clade 2 variants. The resulting reassortant virus strains showed reactivity and cross-neutralizing efficacy similar to the reference serum against H5N1 clade 2 viruses. In addition, mice immunized with the H5N3 mutant produced cross-neutralizing antibodies and cross-protected against distinct H5N1 viral infections. Thus, this strategy, which reduces the need for biosafety level 3 (BSL3) containment facilities, was proven to favor the development of broadly protective vaccines [76,168]. Earlier, He Fang et al. similarly modified the neutralizing epitopes of HA from HPAI H5N1 (A/Indonesia/CDC669/2006) at antigenic sites Sa (155, 156aa) and Sb (189aa) to generate an RG-H5N1 vaccine with a PR8 backbone (RG-EC H5N1 vaccine). Mice immunized with this epitope modified chimeric RG-H5N1 vaccine were found to possess cross-neutralizing antibodies against distinct clades of H5N1 viruses and were protected against lethal challenge by different clades of H5N1 viruses [170]. Another study modified neutralizing epitopes of an existing HA to accommodate circulating H9N2 epitopes to generate a monovalent vaccine with wider protection against H9N2 viruses. Upon identifying the vulnerable positions for divergence through mutant studies, amino acid positions 148, 150 (site I) and 183, 186, 188 (site II) were mutated in the full-length HA gene of H9N2. The BV surface display of the epitope-modified HA rendered cross-protection against H9N2 in a challenge study [77].

### 4.2. Designing Vaccines Based on Conserved Regions

For a broadly protective vaccine to be created, efforts are needed to understand the molecular aspects of the protective antigen with the effects to magnify the humoral response to the target antigen, reduce ‘off-target’ antibody responses and, more importantly, augment the response to the target epitopes [171]. In the case of AIV, the common targets for universal influenza vaccine development consist of the highly conserved stalk domain of the HA protein made up of HA1 and HA2, the ectodomain of the M2 ion channel (M2e) and the internal proteins, nucleoprotein (NP) and matrix protein (M1) [172]. Among these antigens, HA2 holds great potential for developing a universal vaccine against AIVs because its stalk region is not readily accessible to neutralizing antibodies; thus, it faces less selective pressure from the host’s immune system and is highly conserved across genetically distinct strains. The assumption is that the conserved stalk region of HA protein, if targeted, would induce broadly reactive antibodies against influenza viruses within and between multiple strains. To increase the immunogenicity of HA2, HA1 was removed to create a ‘headless HA stalk’ with retention of conformational integrity [173,174]. Alternatively, antibodies against the conserved HA2 stalk can be developed using chimeric HAs (cHAs) that share a similar stalk domain but expresses different head domains (Figure 1C). The stalk domains of these chimeras are made from either H1 or H3 subtypes, and the head domains are derived from the exotic avian influenza virus subtypes to which humans are naïve [175]. The first immunization with a cHA construct primes the response against HA2 and the HA1 globular head domains. HA2 induces a recall response against the stalk domain (Figure 1C) in subsequent booster vaccinations, whereas HA1 triggers a primary response against the antigenically distinct HA1. This cHA approach is platform-independent and can be translated by any downstream practical applications to produce subunit, viral vector or NA based vaccines.

Several studies have also focused on inducing broadly reactive antibodies against both HA head and stalk regions. The conserved amino acids of an immunogenic antigen were first deduced with a phylogenetic analysis of all related virus variants. The conserved amino acids were then designed into a consensus immunogen to produce broadly neutralizing antibodies that neutralize viruses displaying the conserved epitopes. The consensus of the vaccine can range from micro-consensus, whereby the vaccine is designed to protect only against a branch of the phylogenetic tree, to a centralized consensus vaccine, where the antigen is expected to be effective against genetically diverse variants across the phylogenetic tree. However, ancestral and consensus-based conventional antigen designs are intrinsically influenced by the input sequences used to generate the synthetic molecule, and as such, are subject to sampling bias. To overcome these limitations, computationally optimized broadly reactive antigen (COBRA) is used to generate unique immunogens with exclusive spatiotemporal accuracy (Figure 1B). Previous studies have utilized COBRA to generate H5 HA consensus sequences to create novel antigens [176,177]. Numerous studies have since established the protection efficacy from COBRA-generated HA VLPs consensus vaccines. HA VLP-immunized mammals were protected against homologous and heterologous H5N1 lethal challenge, with faster viral clearance and induction of antibody responses against different clades and sub-clades [176,178,179]. A recent study with COBRA HA VLP vaccines effectively induced protective responses against a lethal dose of homologous H5N1 HPAI virus challenge in chickens. However, upon challenge with a genetically diverse H5N1 HPAI virus, COBRA HA VLP vaccines were observed to provide limited protection with a suboptimal reduction in viral load [180]. COBRA-consensus vaccine technology has been combined with antigen display on a live viral platform with promising results [181]. A recent study utilizing an HVT vector displaying COBRA-derived HA VLPs was found to significantly decrease viral shedding and protect against genetically diverse H5 HPAI viruses in challenged chickens. Utilization of an HVT vector also served a dual purpose by protecting against MD and while replicating vaccine to provide a perpetual source of immunogen to induce humoral and cellular immunity to curtail the effect of MDA [108]. A COBRA hemagglutinin (HA) candidate targeting H1 (P1) generated by Sautto et al. [37] was also found to stimulate production of broadly reactive functional antibodies in immunized mice capable of inhibiting HA activity for a wide-ranging panel of H1N1 isolates.

Antigenic cartography (AC) is a new, powerful mathematical and computational method to quantify and visualize fine-grain phenotypic differences among strains of viruses [182]. The technique calculates antigenic distances between influenza field strains and vaccine strains by quantifying raw data for hemagglutination inhibition and microneutralization assays that is interpreted through an intuitive antigenic map. An informed decision is then made on whether the distance is large enough to warrant a vaccine update. Construction of influenza antigenic cartography is available through a webserver [183]. The next critical step is to visualize the HI and neutralization data and to create a map of relationships among the HA proteins of the vaccine strains and the challenge virus. Based on the cartographic selection of field strains for a vaccine candidate, a highly immunogenic LPAI strain of H9, Ck/215, was identified by Wang et al. [184]. Numerous studies have also enlisted cartographic selection for the development of influenza vaccines [185,186]. As an evolving technology, AC possesses several disadvantages, such as oversimplification leading to obscured data. The technology also suffers from bias, and could be skewed by outliers and mapping uncertainties, depending on the quantity of input data. Similar to the sequential vaccination strategy used for cHAs, ‘mosaics’ of immunodominant antigenic sites grafted on the head of HAs (mHAs) (Figure 1D) could be used for sequential immunization. The stalk and the relatively conserved ‘framework’ of the head domain would stay the same, but the immunodominant antigenic sites in the head domain could be varied with every boost (Figure 1D). Potentially, such a vaccination regimen would lead to the induction of anti-stalk antibodies due to the switching out of immunodominant antigenic sites for every vaccination. In addition, antibodies against conserved (immune-subdominant) epitopes in the head domain outside of the classical antigenic sites are also induced and boosted [187].

### 4.3. Development of Vaccine Formulation

Adjuvants can be used in vaccine delivery agents to induce and promote a greater protective immune response against pathogens. They are traditionally divided into two categories. The first comprises immunostimulatory molecules, such as cytokines, Toll-like receptors (TLRs) and bacterial products that enhance and protect the immunogen from degradation in vivo. The second comprises of biodegradable particles such as polymeric nanoparticles, chitosan, liposomes and biomacromolecular compounds like sodium alginate, and gums derived from natural sources. The advantages of biomolecular compounds are that they are non-toxic, biodegradable and possess antiviral properties [188,189,190].

The mucosal site is a popular entry point for multiple viruses, and as such have to be taken into special consideration when it comes to the production of an efficacious and broadly protective viral vaccine against poultry diseases. Mucosal vaccination is the optimal formulation for mass vaccination and currently, there are only a handful of vaccines available for both human and animal applications. Numerous papers have been written on the effects of adjuvants and their unique properties for enhancing mucosal immune responses. Adjuvant for formulation with a broadly protective viral vaccine should ideally stimulate both cellular and humoral responses to induce antigen-specific IgA and systemic IgG [188,189].

Vaccines adjuvanted with TLR agonists or synthetic oligodeoxynucleotides (ODNs) containing unmethylated CpG motifs can bind to TLRs present on immune cells to initiate an immune cascade for the production of proinflammatory and Th1 cytokines. The Th1 immune response is an important mediator for protection against poultry viruses [189]. Another potential adjuvant for enhancing mucosal secretory defense is CpG-NP, a novel avian TLR21 agonist coupled to a poly (lactic-co-glycolic acid) (PLGA)-based hollow nanoparticle platform. Immunization of chickens with a CpG-NP formulated IBV vaccine was found to induce a higher humoral response and stimulate dendritic cell maturation in vivo [176]. Several studies investigating a variety of TLR ligands, such as flagellin, Loxoribine, Pam3CSK4 and the Th1/Th17 adjuvant AddaVax [191] have also produced promising results for their use as effective adjuvants for H5 poultry viral vaccines.

## 5. Conclusions

Amidst the increased demand for poultry meat and products worldwide, commercial and backyard farms are tasked with the continual challenge of producing the all-important supply of animal protein for human consumption. Vaccination is our only line of defense to ensure the demands for poultry products are met worldwide. With the advent of new technology, vaccine design can now be refined through the use of consensus-based algorithms, target conserved epitopes and neutralizing epitopes-based approaches to produce better vaccines against viral pathogens with genetically diverse lineages. Vaccine optimization with techniques such as antigenic cartography also allows for real-time immunological analysis of field and vaccine strains for quicker and more straightforward selection or update of immunogens. Additionally, newer generation of vaccines are being increasingly produced with protective immunogens from multiple species to provide simultaneous protection against co-circulating viral species. Advancement in adjuvant formulation and delivery strategies have likewise seen a shift to mucosal adjuvants for better stimulation of both systemic and mucosal immunity. While many vaccine design strategies for the control of AIV are in place, attention is needed to tackle evolving strains of poultry viruses such as the MDV and NDV. The development of a broadly protective vaccine against MDV would greatly benefit from a combination of improved formulation through the use of a mucosal adjuvant and optimization of vaccine targets based on the growing evidence from experimental studies. Considering the success of new vaccine strategies, more techniques must be channeled into the practical creation of efficacious, safer, and more cost-effective broadly protective viral vaccines.

## Figures and Tables

**Table 1:** Factors involved in the design, development and implementation of poultry vaccines.

**Type of Bird**
Species (chicken, duck, turkey) Sector & Lifespan Broiler: 5–7 weeks Layers: 1–3 years Breeders: 5–7 years
**Nature of Disease**
Extent of spread (episodes, outbreaks, enzootic, epizootic, panzootic, zoonotic, reverse zoonotic) Type of pathogen (viral, bacterial, fungal, parasitic)
**Factors Affecting the Vaccine Response**
Age and status of immune system at the time of vaccination Presence of maternally derived antibodies (MDA) Vaccine storage preparation and administration Duration of immunity Antigenic distance between field virus and the vaccine Immunogenicity of vaccine strain Route of vaccination Intervals and interference between vaccines Immunosuppression Stress, mycotoxins, vaccine induced immunosuppression Cost effective follow-up; differentiating the infected from vaccinated animal (DIVA) strategy
**Types of Vaccines**
Inactivated whole virus Live virus vaccines Attenuated (by traditional serial passaging in embryonated eggs or tissue culture) Reverse genetics Nucleic acid based Recombinant protein subunit, virus like particles (VLPs) Viral vectored Monovalent, bivalent, trivalent or multivalent/chimeric Immune complex
**Vaccine Application (Routes, Number and Frequency of Doses)**
Mass application (spray or drinking water) Individual application (in ovo, eye drop, intranasal, subcutaneous, intramuscular, wing web) Single dose, boosters, multiple doses
